# Proprioceptive disturbances in weightlessness revisited

**DOI:** 10.1038/s41526-023-00318-8

**Published:** 2023-08-11

**Authors:** Uwe Proske, Bernhard M. Weber

**Affiliations:** 1 School of Biomedical Sciences, Monash University, Clayton, VIC 3800 Australia; 2 Institute of Robotics and Mechatronics, German Aerospace Center, 82234 Wessling, Germany

**Keywords:** Physiology, Neuroscience

## Abstract

The senses of limb position and movement become degraded in low gravity. One explanation is a gravity-dependent loss of fusimotor activity. In low gravity, position and movement sense accuracy can be recovered if elastic bands are stretched across the joint. Recent studies using instrumented joysticks have confirmed that aiming and tracking accuracy can be recovered in weightlessness by changing viscous and elastic characteristics of the joystick. It has been proposed that the muscle spindle signal, responsible for generating position sense in the mid-range of joint movement, is combined with input from joint receptors near the limits of joint movement to generate a position signal that covers the full working range of the joint. Here it is hypothesised that in low gravity joint receptors become unresponsive because of the loss of forces acting on the joint capsule. This leads to a loss of position and movement sense which can be recovered by imposing elastic forces across the joint.

## Introduction

There can be no more vivid example of the effect of weightlessness on human limb position sense than the observation made by Schmitt and Reid, as described in Lackner and DiZio^1^; “Astronauts and cosmonauts have reported that on awakening in the dark (during flight), they may not feel where their arms are, or if they see a luminous watch dial, they may not immediately realise it is on their wrist.” Such observations point to a significant disturbance of limb position sense under conditions of weightlessness.

On rare occasions, subjects present in the clinic who have lost their sense of position. Someone unfortunate enough to have such a loss is initially quite helpless^2^. It has been speculated that the sense of position is a fundamental aspect of one’s self-awareness, the “sense of self”^2,3^ and without it we are lost. Here, however, we should keep in mind the extraordinary adaptability of chronically deafferented human subjects, for example, in tasks such as making reaching movements across unexpected force fields^4^.

We have recently reviewed the topic of proprioception and, specifically, human limb position sense, under conditions of weightlessness^5^. Our ideas were based on changes in discharges of muscle spindles, the peripheral sensors believed responsible for position sense. Here we have re-visited the subject since there is some uncertainty about the existence of ongoing fusimotor activity to spindles in a relaxed limb. If so, it throws into doubt the hypothesis for a decrease in fusimotor drive in microgravity^6^, as well as the linked explanation for the action of elastics (Bringoux et al. ^7^, p. 2545). We have concluded that if these studies are going to define our understanding of the subject, at the very least, more experiments will be required to help strengthen their case. In addition, we have put forward our own, new hypothesis. It, too, will require further verification, but it has the advantage of providing a fresh and rather different outlook on the subject. Perhaps the novelty of the proposal will help to revitalise the debate about the origin of this important proprioceptive sense in an era where space travel is becoming progressively more frequent.

The studies of Lackner and DiZio^6^ and of Bringoux and his colleagues^7^ have been considered in detail in the Results section below, under the subheadings, “Kinaesthetic illusions in responses to muscle vibration” and “Effects of stretching elastic straps across the joint”. Then, we discuss “The sense of movement under conditions of weightlessness” and report on a recent series of studies in the section “Measuring kineasthetic acuity with an instrumented joystick”. They are followed by our earlier proposal relating to the influence of “muscle thixotropy” and, finally, consideration of yet one more possible factor, under the heading, “Is kinaesthetic sensibility reduced in low gravity due to lack of input from joint receptors?”

## Results

### Kinaesthetic illusions in response to muscle vibration

The effects of muscle vibration on the senses of limb position and movement, under conditions of weightlessness, were first described by Lackner and DiZio^6^. They measured vibration evoked illusions of position and movement in elbow muscles during a series of parabolic flights. The illusions perceived in the vibrated arm, which was held fixed in position, were indicated by the other arm. For biceps vibration, they found that the mean changes in perceived position of the forearm during horizontal flight (1 g) was 16.6° in the direction of elbow extension, compared with 2.9° in 0 g and 31.6° in 1.8 g, both in the direction of extension. Vibration of triceps produced similar, although smaller, errors into flexion. In reviewing their findings Lackner and DiZio^6^ concluded that tonic vibration reflexes (TVR) in the vibrated muscle showed g-related effects during parabolic flight, being diminished during the low gravity phase and enhanced in the high gravity phase. They interpreted this result in terms of spindle vibration sensitivity, modulated by gravity-dependent changes in fusimotor activity; it was reduced in 0 g and enhanced in 1.8 g and one way this could be achieved was through alterations in otolith-spinal influences.

There were two ways in which the fusimotor activity could be generated; it could be part of the alteration in postural tonus triggered by the changes in g-level. Mechanisms which are responsible for changes in tonus are believed to involve stimulation of the otoliths, leading to activation of both skeletomotor and fusimotor neurons via the vestibulospinal pathway. Alternatively, the vestibulospinal influences could act through the TVR initiated in the vibrated muscle. However, in a study of spindle responses to vibration during muscle contractions, Burke et al. ^8^. concluded that the TVR operates predominantly, or exclusively, on alpha motoneurons, without engaging fusimotor neurons. Therefore, there remains some uncertainty about this second mechanism. For both of these explanations the implication is that when a muscle is vibrated at 1 g, there is ongoing fusimotor activity. This is a point of controversy^5^.

Since in the experiments of Lackner and DiZio^6^ the arm was held supported, fixed in position, it was presumably relaxed during vibration. It remains uncertain whether under the conditions of this experiment a TVR was always generated. A vibration illusion can occur equally well in the absence of any reflex or postural contraction^9^. It raises the question of whether there is any ongoing fusimotor activity in relaxed muscle. The majority view appears to be that if a muscle is fully relaxed, there is no ongoing fusimotor activity^10^ (but see Banks et al. ^11^, p. 2349). If there is no fusimotor activity during vibration at 1 g, it undermines the argument that spindle gain attenuation through a reduction in fusimotor activity is responsible for the loss of position sense in weightlessness^1^. In the future it would be well worth repeating the experiments of Lackner and DiZio^6^ and to determine whether under the conditions of their experiment a TVR was always generated and whether or not this was accompanied by an increase in fusimotor activity.

### Effects of stretching elastic straps across the joint

Intriguingly, the explanations provided by this second set of experiments on position sense in weightlessness are rather different from those provided by Lackner and DiZio^6^. They, therefore, raise some uncertainty over what is actually going on. The first reported use of elastics was by Roll et al. ^12^. During spaceflight, blindfolded subjects were asked to stand upright with vibrators attached to their ankles. Both whole-body postural responses and movement illusions were studied during vibration of ankle dorsi and plantar-flexors. This experiment is based on the well-known observation that paired vibration of Achilles tendons of both legs leads blindfolded subjects to perceive themselves as leaning forwards. To compensate, they lean backwards, in the process sometimes falling over.

During vibration of ankle muscles under conditions of weightlessness, both postural responses and illusions of position and movement were attenuated. When elastic straps were stretched between the subject’s jacket, and the footrests, the load this imposed on the ankles was of gravity-like downward forces (50 kg) exerted parallel to the long axis of the body. To resist these forces and maintain an erect posture, the subject had to lock their knees and increase their leg muscle activity. Under these conditions there was a recovery of normal kinaesthetic illusions.

To account for their findings the authors concluded that the terrestrial internal model of postural regulation of proprioceptive origin seems to persist in microgravity and that it can be readily recalled by means of elastics; these provide astronauts with earth-like sensory and motor information, as a result of the external forces generated, simulating the effects of gravity.

In a similar approach, Bringoux and colleagues^7^ applied elastic straps to subjects’ arms to mimic gravity-like torque at the shoulder joint. In an arm reaching task towards pre-defined angular positions during parabolic flight, blindfolded subjects overshot the target during hypergravity and undershot it during microgravity. In microgravity, adding the gravity-like torque with the elastics allowed subjects to perform as well as during normogravity, both in terms of accuracy and movement kinematics.

In order to account for their results, Bringoux et al. ^7^. provided two possible explanations. In one, it was proposed, that the presence of the elastic straps led to a modification of discharges in muscle spindles as a result of bearing the load, thereby improving proprioceptive acuity. Here the suggestion is that the necessary increase in alpha motoneuron activity required to counteract the gravity-like torque produced by the elastics, was accompanied by enhanced fusimotor activity^10^, sufficient to maintain spindle sensitivity to muscle length changes. In an alternative explanation, the extra motor command required to overcome the elastic load may have given rise to a greater sense of effort which was able to make an additional contribution to position sense^13^.

### The sense of movement under conditions of weightlessness

When a muscle is vibrated, it generates illusions of both movement and displaced position. In fact, the predominant sensation is one of movement. In their account of effects of gravity changes on kinaesthesia, Lackner and DiZio^6^ focussed their attention on limb position sense. However, they did comment on movement sense; “The velocity sensations were consistently reported to be faster in 1.8 g and slower in free fall than in 1 g.”

The sensory origins of movement and position sense are not identical; position sense is generated, based on signals coming from both the primary and secondary endings of muscle spindles. Movement sense, on the other hand, is generated predominantly by the primary endings of spindles^11^. The high dynamic sensitivity of primary endings is responsible for the strong illusion of movement generated during vibration.

In their experiments during parabolic flight, Bringoux et al. ^7^. reported perceived changes in position sense with gravity. However, they also analysed the kinematics of the reaching movements. They found arm kinematics similar in 1 g and 0gE (presence of elastics in low gravity conditions). The result suggested that gravity-like arm loading in weightlessness with elastic straps helps preserve the organisation of motor commands established in 1 g. Specifically, the temporal structure of the movements was similar in 1 g and 0 gE.

According to current models of motor control, afferent signals that arise from self-generated movements are inhibited by a mechanism that compares predicted and actual sensory feedback. In other words, input to the control of a movement comes not only from the afferent signals provided by proprioceptors, but by motor programs stored centrally and established, based on past experience. If there is a large difference in the sensory consequences predicted by the motor program and what is actually fed back from the periphery, the comparison mechanism is unable to operate. Bringoux et al. ^7^. speculated that this had happened at 0g and 1.8 g because of the mismatch that occurred between the expected and actual sensory feedback during the changed gravity conditions; the afferent rate was higher than expected at 1.8 g and lower at 0 g. Such a mismatch would not be present in 1 g or in 0 gE. This explanation would therefore accord with the conclusion of Roll et al. ^12^. that elastics allow recall of internal motor programs laid down at ground level. It also provides a link between the disturbance of position and movement sense in low gravity; the presumed low rate of afferent activity, responsible for disturbance of position sense, also interferes with the sensory matching mechanism during movements.

### Measuring kinaesthetic acuity with an instrumented joystick

**Fig. 1:**
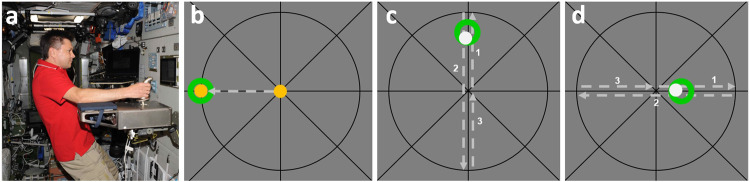
Aiming and Tracking Experiments. **a** Moving the joystick onboard the ISS. **b** The static target screen showing green targets and yellow aiming cursor. The task was to place the aiming cursor in the centre of the selected target. Vertical and horizontal tracking task (**c, d**): the target to track (green) moved from the centre to the top of the target screen, or from the centre to the right (1), then reversed, passing through the centre (2) and then reversed again to return to the centre (3). The task was to try to keep the grey aiming cursor within the target throughout the movements.

The effects of elastics in weightlessness were also investigated in a recent series of experiments^14–17^ utilizing a different type of positioning task. In a computer-based simulation, static and dynamic target positions had to be reached or tracked with a cursor which was controlled by a force-reflecting joystick (see Fig. 1a). The static targets (b) were located on the horizontal and vertical axes of a crosshair, while tracking movements (c, d) were also performed along these axes. Hence, joystick motions used to track the target had to be performed in both the transverse and sagittal planes.

In an isotonic baseline condition, the joystick could be moved freely without any restriction and without any significant counterforces (like friction). In addition, the motors of the joystick allowed the simulation of various mechanical properties: elastics (a re-centering spring), viscous damping and raising the mass of the joystick’s handle. Aiming and tracking performance was investigated, making use of these changing properties under conditions of simulated weightlessness (during shallow water immersion^14^) as well as in weightlessness during spaceflight (onboard the ISS^15–17^). The authors reported that in (simulated) weightlessness, longer times were required for locating the cursor within the static targets. In addition, performance in the movement tracking task was poorer in the isotonic condition. During spaceflight this effect was most obvious during the initial phase of adaptation to microgravity (2 weeks of exposure), and performance then improved during the subsequent experimental sessions (4 and 6 weeks of exposure). The overall impression was that there was a rapid loss of accuracy in both static placement of the cursor as well as in movement tracking accuracy following exposure to microgravity.

These disturbing effects by microgravity on proprioception could be partially offset by the choice of appropriate mechanical characteristics for the joystick. For example, even low levels of viscous damping led to improved tracking performance. The most probable explanation for this outcome was that subjects were exerting voluntary force to move the joystick, which led to co-activation of fusimotor fibres to the spindles, to recover spindle sensitivity^10^. Here it should be kept in mind that the fusimotor recruitment would last only for the duration of the contraction and there is evidence that static fusimotor fibres are recruited, which will tend to lower the dynamic sensitivity of spindles^18^.

An unexpected finding was that low to moderate spring stiffness also had a positive effect, both on tracking and aiming precision^15–17^. However, this effect was only evident for tracking motions in the transverse plane. Re-analysing the aiming motions showed a similar direction-dependency. A possible explanation for this finding related to the biomechanical requirements for the two directions of movement. While sagittal movement of the joystick required a rotation of the wrist and, to a lesser extent, shoulder and elbow rotations, the transverse movement required a rotation of the entire forearm (pro- and supination). Therefore, biomechanical stabilization of transverse motion was generally more demanding and required more accurate feedback about limb position compared with sagittal motion. In the transverse movements, the joystick is rotated to the side and the weight of the forearm accordingly shifts, creating a torque acting on the arm. This torque, as a source of information for determining arm position, is lost in weightlessness.

It is remarkable that spring stiffness—which had no positive effect at all under terrestrial conditions—led to a restoration of positional accuracy in weightlessness. And this was the case despite the fact that the torque generated by the spring acted in the opposite direction compared to the torque generated by gravity. The general conclusion from these studies during spaceflight, supported the idea of an improvement in proprioceptive acuity whenever torque was exerted on the relevant joints by the chosen intervention.

### The influence of muscle thixotropy on muscle spindle signalling

We have previously contributed to the debate about the origins of the decline of position sense under conditions of weightlessness^5,19^. We have proposed that one contributing factor to the reduction of position sense acuity in a gravity-free environment is the influence of thixotropy^20,21^. Muscle spindles show thixotropy because the intrafusal fibres on which the sensory endings lie are striated muscle. All striated muscle shows thixotropy^21^.

In a gravity-free environment, movement of the arm will be initiated by weak, brief contractions of arm muscles. Because the influence of gravity is missing, resting muscle tone is reduced and during most of the movement, arm muscles are likely to remain passive. Whenever a limb is moved while its muscles remain passive, the movement will introduce slack in muscle fibres and spindles of those muscles undergoing shortening during the movement. After repeated forwards and backwards movements, the amount of slack in arm muscles is likely to grow, leading activity in spindles of most arm muscles to fall to low levels. Evidence for the presence of slack in spindles is the effect of a muscle contraction, “post-contraction sensory discharge”^22^. The contraction will take up any slack and thereby raise spindle discharge levels. In the presence of slack, the signal required for monitoring muscle length, and which provides the afferent basis for position sense^23^, would be greatly weakened. Stretching elastics across the joint would lead to an increase in voluntary force required to maintain a chosen arm position. This would involve alpha-gamma co-activation^18^, which would reduce any slack present and help to recover spindle sensitivity.

To conclude, the recovery of near-normal kinaesthetic responses with the use of elastics has several possible explanations; in order to overcome the force generated by the elastic bands, the subject generates a voluntary contraction which engages the fusimotor system to raise spindle sensitivity and recover kinaesthetic acuity. In a second explanation, the force exerted by the elastic bands, “recovers gravitational information provided by joint torque”^7^. We propose that this is achieved by the force of the elastics acting on the joint capsule to engage joint receptors and, in the process recover a near-normal position sense. Finally, the effect of elastics on movement kinematics suggests that the unexpected afferent feedback encountered in high and low gravity conditions interferes with movement program selection. While we believe that muscle thixotropy plays a role in degrading the kinaesthetic senses in weightlessness, its effect does not seem large enough to be able to account for the complete loss of this sense.

### Is kinaesthetic sensibility reduced in low gravity due to lack of input from joint receptors?

The ideas presented here are based on the observations that in 0g, applying elastics to the limb, while it maintained position, could recover normal position sense acuity^7,14–17^. It is hypothesised that the primary effect of elastic forces is on joint capsule receptors whose afferent discharge contributes to the position sense signal. The absence of joint input modifies the position signal and interferes with selection of motor programs for movement sense.

**Fig. 2:**
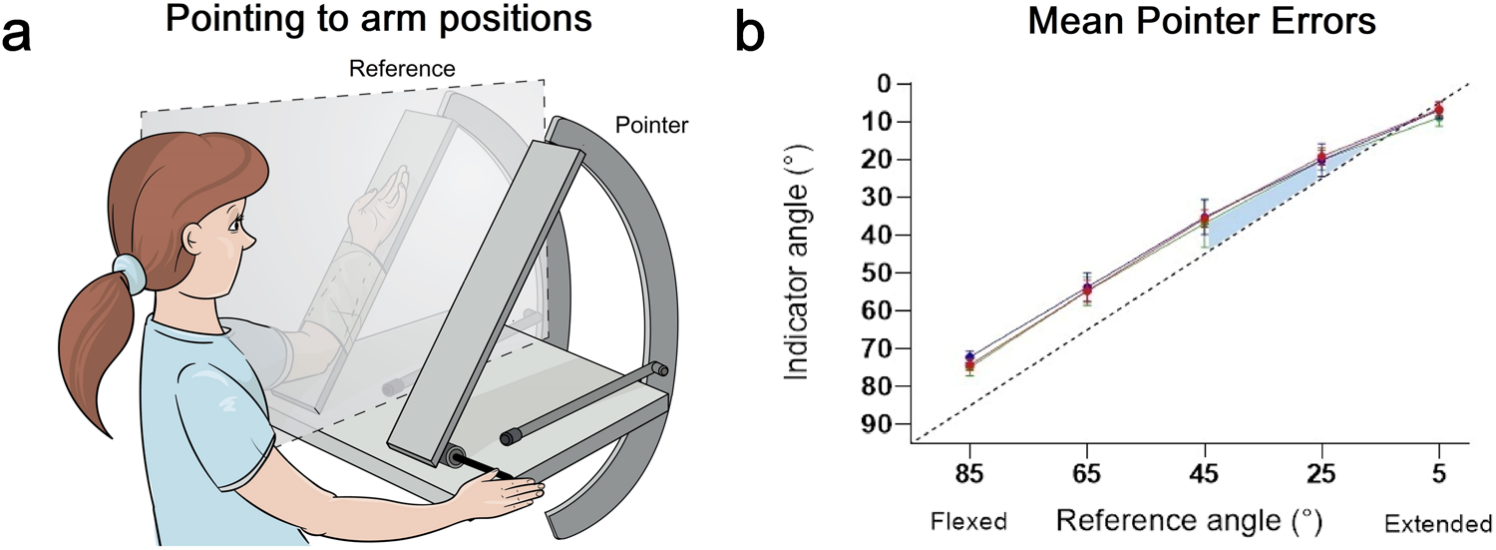
Position Pointing Task. **a** The participant’s reference arm was strapped to a paddle, hidden behind a screen. With their other arm they moved the pointer paddle, rotating it by means of a lever at its base. Both paddles had potentiometers at their hinge joints to provide a continuous output of elbow angle. The task was to move the pointer paddle to a position where it was perceived to be in line with the hidden reference forearm. **b** Plot of the angle indicated by the pointer paddle against the position of the hidden reference forearm. Position errors measured at each of a range of test angles between 90° (forearm vertical to supporting base) and 0° (forearm fully extended). Dashed line, reference and indicated positions are identical. Values above the line, errors into extension. At each test angle, the reference arm was subjected to three different thixotropic conditionings, shown in red, green or blue. For each colour the plotted value represents the mean ( ± SD) of three repeated measurements at that angle, pooled for 10 participants. The shaded portion of the angle range indicates where position errors became progressively smaller. Figure modified and redrawn from Chen et al. ^24^.

We have recently been studying position sense at the human forearm in a position pointing task^24^. Subjects had one arm sitting, hidden behind a screen and with their other arm they had to point to the perceived position of the hidden arm (see Fig. 2).

Position sense was measured over a wide range of elbow angles; Across the mid-range of angles (85°–45°, Fig. 2b) a consistent error of position sense, in the direction of extension, was observed. That is, the subject pointed to a position about 10° further in the direction of extension than the actual position of the arm. The source of these errors is probably a bias in the muscle spindle signal, favouring a larger level of activity coming from elbow flexor spindles compared with extensor spindles. A larger flexor spindle signal would make the subject think their arm was more extended than was really the case.

The observation relevant to the present account was a reduction in the extension errors for test angles that approached the limit of arm extension (45°–5°, shaded area Fig. 2b), with an error close to zero at a test angle of 5°. To explain such a trend, it is necessary to postulate a source of signal that is able to indicate joint angles approaching the limit of the movement range. Slowly adapting joint receptors do that. They are able to be engaged at both the limit of flexion and extension of the arm^25^. Our working hypothesis was that in the mid-range of joint angles, position was signalled predominantly by muscle spindles^21^. In our pointing study, as the arm was moved to test angles approaching full extension, the activation angle for some joint receptors would be reached and they would begin discharging. In other words, at the more extended angles discharges would be generated in both spindles and joint receptors. We propose that it was the action of joint receptors which was responsible for reducing position errors at extended angles. Our data suggest that over about 30% of the total angle range at the elbow, some influence of joint receptors would be expected. For a detailed discussion, see Proske^26^.

In a series of vibration studies, Craske^27^ was able to evoke sensations of hyperextension of the forearm by slowly extending it while vibrating elbow flexors, or sensations of hyperflexion by slowly flexing it while elbow extensors were vibrated. Muscle vibration is a means of selectively stimulating muscle spindles. Craske postulated that the normal spindle discharge rate: muscle length relation, encountered in everyday activities, was extrapolated by the brain to account for the much higher discharge rates evoked by vibration, to arrive at length values representing joint overextension or overflexion. The important conclusion from these observations is that discharges of spindles do not contain any information alerting the brain of the approaching limit in the working range of the joint. That, we propose, is the role of joint receptors. In other words, while spindles faithfully signal muscle length and therefore joint angle in the mid-range of joint angles, they are unable to signal the limits of joint movement.

Here it is proposed that at some point in the brain, the spindle and joint receptor signals would be combined to generate a composite which incorporated values of limb position over the whole working range of the joint, up to its anatomical limits. Such an interpretation would explain the declining position errors in the position pointing study for test angles where the forearm was approaching its limit of extension^24^.

There is some evidence in the literature about signals generated during movements made at a joint, suggesting that as part of the central processing of joint information there is a combination of a mid-range signal (spindles) and a joint limit signal (joint receptors). Poggio and Mountcastle^28^, recorded from third-order neurons in the ventrobasal complex of the thalamus in monkeys, driven by contralateral joint inputs. Each neuron responded to rotation of the joint in one direction only. Its discharges reached their maximum either at full flexion or full extension of the joint and the range of excitatory angles for these neurons was wide, four times wider than had been reported for joint receptors. We would add that for a neuron responding to joint extension, the mid-range spindle signal from stretched flexor muscles would combine with activity in extension sensitive joint receptors to produce the characteristics of the thalamic neuron. The same would occur with flexion sensitive thalamic neurons; they represent a combination of an extensor spindle signal with activity in flexion sensitive joint receptors.

What does all of this have to do with the loss of position sense under conditions of weightlessness? Slowly adapting joint receptors (Ruffini endings) lie within the joint capsule. They are the most likely candidates for joint position sensors. The stimulus for these receptors is stretch or compression of the capsule^29^. Only at the extremes of the movement range is the joint capsule stretched; it can also be distorted by stretch or contraction of adjacent muscles^30^. For a relaxed arm in the extended position, under conditions of 1 g, the weight of the forearm will bear down on the elbow joint, imposing a torque on the joint capsule to initiate activity in extension-sensitive joint receptors. Similarly, for the fully flexed forearm, the weight of the forearm bearing down on the upper arm exerts compressive forces to excite flexion-sensitive joint receptors. It follows that if the arm becomes weightless, there will be no torque acting on the joint and therefore no joint receptor activity would be initiated.

In envisaging the spindle-joint receptor interaction, over the working range of the joint it is necessary to see the spindle signal as an incomplete position signal. That is, in weightlessness, the absence of a joint receptor input prevents the central processing involving integration of spindle and joint signals, leading to generation of a faulty position signal. The lack of defined limits in that signal, representing the limits of joint movement, makes the subject unsure of the actual position of their arm. Applying an elastic with the appropriate extension characteristics across the joint would be expected to reintroduce forces on the joint capsule to recruit joint receptor activity. As a result, the integration of joint receptor and spindle signals would become possible again, allowing computation of a full position sense signal.

In the literature there are few examples of measurements of position sense in the absence of joint receptor input. It has been reported that at the knee, infiltrating the knee joint with local anaesthetic did not affect position sense in the mid-range of joint movement^31^. Position sense towards the extremes of joint movement was not tested, so a contribution from joint receptors was not expected. In a similar experiment, but where position of a finger was held close to its limit of flexion or extension, anaesthesia of the proximal interphalangeal joint led the subject to perceive position of the finger in its mid-range of movements^32^. The shift in perceived position of the extended finger was 25° into flexion and for the flexed finger it was 25° into extension. Therefore, removal of joint input generated large position errors, although some mid-range signalling capacity had been preserved. In discussing their findings, the authors proposed that in the presence of a digital nerve block the zero rate of firing of joint receptors was meaningful to the brain, which interpreted the incoming signal as the finger being at, or near, its mid-range. In other words, the lack of joint input interfered with calibration of the position signal.

Such a conclusion accords with the findings of Gooey et al. ^33^. who rendered the subject’s arm essentially weightless by counterbalancing the weight of the arm. It was found that position sense accuracy remained unchanged, but the standard deviation of position errors increased significantly. It suggested that with a counterbalanced arm subjects had become less sure of its position.

The idea of joint receptors as joint limit detectors is not new^34,35^. What is new in our proposal is the concept of an integrated spindle-joint receptor signal as essential for computing limb position sense over the full range of joint movements. The withdrawal of one or other of these two components can lead to disruption of the position signal. The simplest way to do that is to remove the force of gravity thereby reducing forces acting on the joint capsule.

Susceptibility to changes in responsiveness in 0 g is not necessarily restricted to joint receptors. Tendon organs are muscle force-sensitive receptors which would be expected to respond to any changes in forces in muscles in 0 g, as well as from introducing elastics. However, because they are believed not to participate in the generation of position sense^23^, they are unlikely to contribute to the disturbance of kinaesthesia in weightlessness.

If we are right, and the position signal is altered by the lack of joint input in a weightless environment, it is worth speculating whether there are other consequences. There is evidence that signals from muscle receptors contribute to the process of embodiment, the acquisition of a body part as one’s own^36^. It would be interesting to know whether astronauts experience uncertainty over body ownership in the absence of vision. The example described earlier of the astronaut being unaware of the watch on his wrist is suggestive. Another, more long-term, consequence after periods of weightlessness is having difficulties with the laying down of spatial memories^5,37^. Our working hypothesis is that proprioceptors access a map in the brain, the body model^38^. Here joint angle signals provided by proprioceptors are combined with information about the size and shape of body segments to allow perception of position of the body in external space. If proprioceptive signals are defective due to the absence of joint afferent input, their access to the body model may be compromised and that could interfere with the laying down of spatial memories. These ideas are worthy of study in future experiments.

## Discussion

The kinaesthetic senses, the senses of limb position and movement, are both disturbed under conditions of weightlessness. An experimental manipulation that allows recovery of kinaesthetic acuity in low gravity is to stretch elastic straps across the joint under study. It is proposed here that the forces exerted by the straps on the joint capsule sensitise joint receptors. Muscle spindles, by themselves, are unable to signal joint position over the full working range of a joint; it requires input from joint receptors to be able to do that. It is proposed that this requirement underlies the disturbance of position sense in weightlessness. In low gravity, the spindle signal, deprived of joint input, also leads to a disturbance of movement kinematics by interfering with the matching process between self-generated afferent signals and their prediction by motor commands. That, in turn, changes the kinematics of a movement and leads to errors in reaching and tracking tasks.

We have chosen not to speculate on the time-course of effects due to our proposed mechanism, since we did not study it. However, it is likely that the onset will be prompt, arising as soon as gravity levels have fallen significantly and recovery, on return to Earth, should be similarly rapid, since the mechanism relies entirely on forces acting on joint capsules.

Another important question concerns possible remedial action the astronaut might undertake, such as exercise, to minimise proprioceptive losses under conditions of weightlessness. In studies of effects of exercise on position sense (Proske^19^, p 2454–2455) it was concluded that the observed effects were not of a peripheral origin, but arose within the central nervous system. While the effect of exercise on proprioception in 0 g has not been tested by us, it seems unlikely that this is able to influence the forces required to act on joint capsules to recover joint receptor responsiveness.

### Reporting summary

Further information on research design is available in the Nature Research Reporting Summary linked to this article.

### Supplementary information

Reporting Summary

## Data Availability

Data sharing is not applicable to this article as no datasets were generated or analysed during the current study. The data cited is all contained in the articles of Weber and colleagues^14–17^ and Chen et al. ^24^.

## Code availability

Code sharing is not applicable to this article as no datasets were generated or analysed during the current study.
